# Supplementary material for: MetaRibo-Seq measures translation in microbiomes
Source: Nat Commun. 2020 Jun 29;11:3268. doi: 10.1038/s41467-020-17081-z (PMC7324362; doi:10.1038/s41467-020-17081-z)
Supplement: Supplementary file 10 — Supplementary Data 7 [file 41467_2020_17081_MOESM10_ESM.zip › File2/Confidence_High_Taxonomy/146827_out.krona.html]

Javascript must be enabled to view this page.

members
magnitude
magnitudeUnassigned
count
unassigned
taxon
rank

146827\_out

2

2
superkingdom
2

2
phylum
201174

class
1760
2

2037
order
1

family
2049
1

1
76833
genus

712888
species
1

SRS104093\_contig\_number\_9420

1
order
85006

1
145360
family

60919
genus
1


SRS149328\_contig\_number\_contig-100\_13834.49031
60920
species
1
